# TRP channels: sensors and transducers of gasotransmitter signals

**DOI:** 10.3389/fphys.2012.00324

**Published:** 2012-08-09

**Authors:** Nobuaki Takahashi, Daisuke Kozai, Yasuo Mori

**Affiliations:** ^1^Laboratory of Molecular Biology, Department of Synthetic Chemistry and Biological Chemistry, Graduate School of Engineering, Kyoto UniversityKyoto, Japan; ^2^Advanced Biomedical Engineering Research Unit, Kyoto UniversityKyoto, Japan; ^3^CREST, Japan Science and Technology AgencyChiyoda-ku, Tokyo, Japan; ^4^Laboratory of Environmental Systems Biology, Department of Technology and Ecology, Hall of Global Environmental Studies, Kyoto UniversityKyoto, Japan

**Keywords:** TRP channels, gasotransmitter, nitric oxide, oxygen, TRPC5, TRPC6, TRPV1, TRPA1

## Abstract

The transient receptor potential (*trp*) gene superfamily encodes cation channels that act as multimodal sensors for a wide variety of stimuli from outside and inside the cell. Upon sensing, they transduce electrical and Ca^2+^ signals *via* their cation channel activities. These functional features of TRP channels allow the body to react and adapt to different forms of environmental changes. Indeed, members of one class of TRP channels have emerged as sensors of gaseous messenger molecules that control various cellular processes. Nitric oxide (NO), a vasoactive gaseous molecule, regulates TRP channels directly *via* cysteine (Cys) S-nitrosylation or indirectly *via* cyclic GMP (cGMP)/protein kinase G (PKG)-dependent phosphorylation. Recent studies have revealed that changes in the availability of molecular oxygen (O_2_) also control the activation of TRP channels. Anoxia induced by O_2_-glucose deprivation and severe hypoxia (1% O_2_) activates TRPM7 and TRPC6, respectively, whereas TRPA1 has recently been identified as a novel sensor of hyperoxia and mild hypoxia (15% O_2_) in vagal and sensory neurons. TRPA1 also detects other gaseous molecules such as hydrogen sulfide (H_2_S) and carbon dioxide (CO_2_). In this review, we focus on how signaling by gaseous molecules is sensed and integrated by TRP channels.

## Introduction

**Figure 1:**
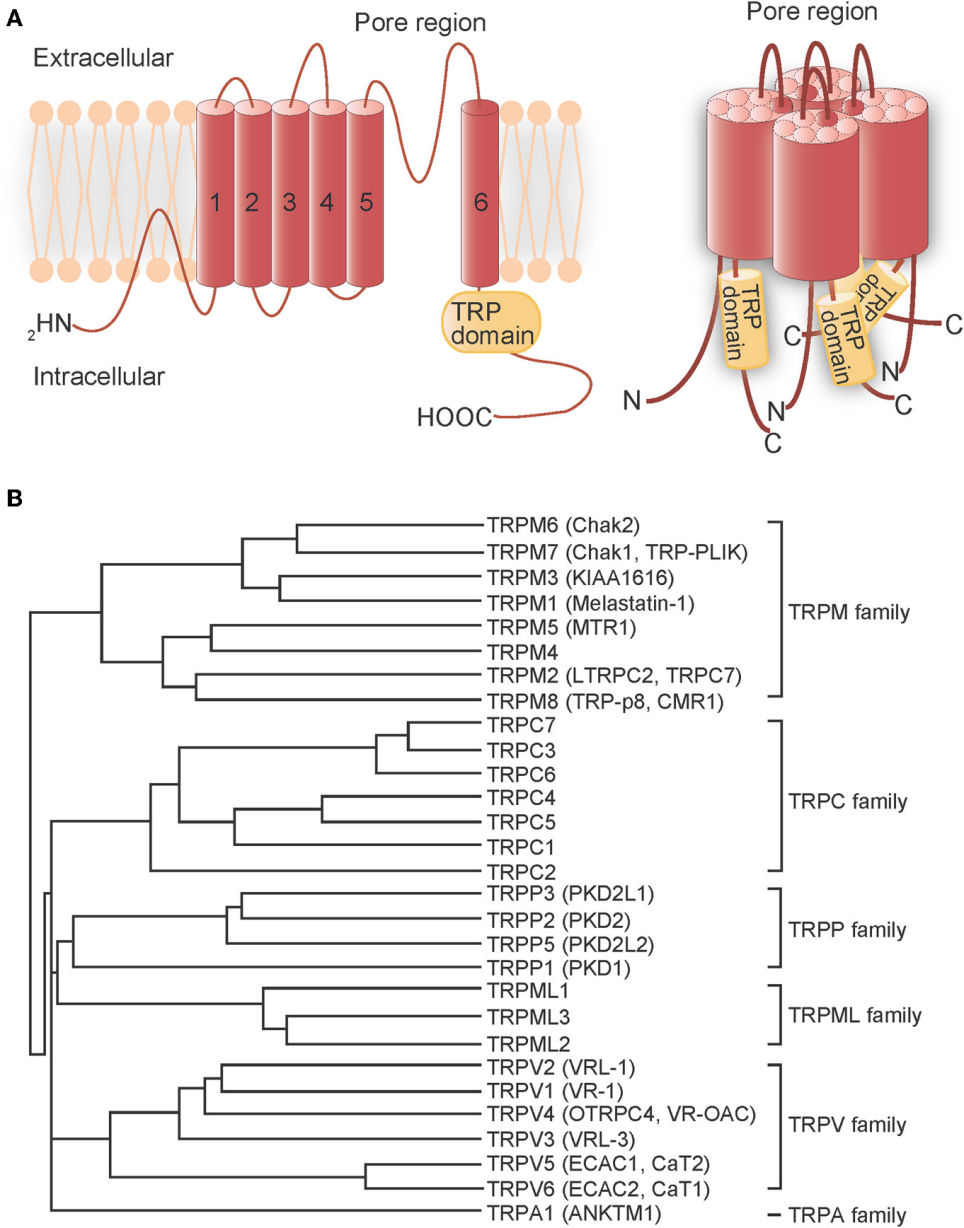
**Transmembrane topology and phylogenetic tree of mammalian TRP channels. (A)** Transmembrane topology (left) and the quartenary structure of TRP channels (right). The TRP protein has six putative transmembrane domains, a pore region between the fifth and sixth transmembrane domains and a TRP domain in the C-terminal region. The TRP protein assembles into homo-tetramers or hetero-tetramers to form channels. **(B)** Phylogenetic tree of mammalian TRP channels based on their homology.

Transient receptor potential (TRP) proteins are the product of *trp* genes, which were first discovered in *Drosophila melanogaster*, but later found to have homologs in other species. These gene products form cation channels that detect and transduce cellular stimuli into electrical signals (*via* changes in membrane potential) or chemical signals [*via* changes in intracellular Ca^2+^ concentration ([Ca^2+^]_i_)] (Montell et al., 2002; Clapham, 2003; Voets et al., 2005). TRP proteins are putative six-transmembrane domain polypeptide subunits that assemble into tetramers to form channels (Figure 1A). In mammalian systems, TRP channels comprise six related protein subfamilies: TRPC, TRPV, TRPM, TRPA, TRPP, and TRPML (Clapham et al., 2005) (Figure 1B). The TRPC homologs form receptor-activated Ca^2+^-permeable cation channels (RACCs) that, when activated by receptor stimulation, induce phospholipase C (PLC) to hydrolyze phosphatidylinositol-4,5-biphosphate (PIP_2_) into inositol-1,4,5-trisphosphate (IP_3_) and diacylglycerol (DAG) (Zhu et al., 1996; Vazquez et al., 2004). Store-operated channels (SOCs), which are activated by IP_3_-induced Ca^2+^ release and depletion of endoplasmic reticulum (ER) Ca^2+^ stores, can also be categorized as RACCs. In contrast, TRPV Ca^2+^-permeable channels can be functionally defined as thermosensors (Caterina et al., 1997; Clapham, 2003; Patapoutian et al., 2003; Clapham et al., 2005). TRPV1, originally identified as the receptor for the vanilloid compound capsaicin, is responsive to heat (>43°C); proton (H^+^) concentration (pH < 5.6); the intrinsic ligand, anandamide; and receptor-driven PLC activity (Clapham et al., 2005). High temperature also activates TRPV2 (>52°C), TRPV3 (>31°C or >39°C), and TRPV4 (>27°C). TRPV5 and TRPV6 comprise a different subfamily because they are activated by [Ca^2+^]_i_ (Clapham, 2003; Clapham et al., 2005). The TRPM subfamily is named after melastatin (TRPM1), a tumor suppressor protein isolated in a screen for genes whose level of expression is inversely correlated with the severity of metastatic potential of a melanoma cell line (Duncan et al., 1998), and contains eight mammalian members. TRPM8 channels, in contrast to TRPV channels, are activated by low temperatures (<25°C) and menthol (McKemy et al., 2002; Peier et al., 2002). The sole member of the TRPA subfamily, TRPA1, has a large N-terminal domain with 17 predicted ankyrin repeat (AnkR) domains (Gaudet, 2008). Pungent compounds, such as the allyl isothiocyanate found in mustard oil, trigger TRPA1 activation (Jordt et al., 2004). TRPA1 has been shown to respond to noxious cold (<17°C), although its activation by cold remains controversial (Story et al., 2003; Bandell et al., 2004; Jordt et al., 2004; Nagata et al., 2005; Bautista et al., 2006; Kwan et al., 2006; Sawada et al., 2007; Karashima et al., 2009). Thus, TRP channels serve as sensors for a variety of environmental factors. With the exception of TRPM4 and TRPM5 (Nilius, 2007), all of these TRP channels have some Ca^2+^ influx activity that is a component of their regulation of diverse cellular processes.

Gaseous molecules, such as oxygen (O_2_), nitric oxide (NO), carbon monoxide (CO), hydrogen sulfide (H_2_S), and carbon dioxide (CO_2_), have been shown to play important roles in biological signal transduction. These molecules share several unique physicochemical properties and exert their biological activities through mechanisms distinct from those of other signaling molecules as summarized by Suematsu (2003). Firstly, these molecules are highly membrane-permeable and can readily convey signals in an autocrine, paracrine and/or juxtacrine manner. Secondly, they exert their biological actions *via* a variety of interactions with macromolecules: covalent binding of gases to prosthetic metal complexes in receptor proteins; non-covalent binding to the regulatory subunits of proteins; and space occupancy in and around the protein structure that impedes the access of other gases to the functionally critical protein motifs. Recent evidence suggest that NO, CO, H_2_S, and CO_2_ function as signaling molecules that also play critical roles in mediating the biological effects of changes in O_2_ availability (Semenza and Prabhakar, 2012).

Among the gaseous signaling molecules, NO is the most extensively studied. Its biological significance and the systems by which it is generated were first revealed in the 1980s (Furchgott and Zawadzki, 1980; Palmer et al., 1988; Sakuma et al., 1988) and it is now known to regulate a variety of biological events, including vascular relaxation and neurotransmission. Conversely, excessive generation of NO and NO-derived reactive nitrogen species (RNS) has been implicated in a number of pathological conditions (Reiter, 2006). Cyclic GMP (cGMP) is the canonical mediator of NO signaling. However, the importance of a cGMP-independent signaling pathway involving protein S-nitrosylation is becoming increasingly recognized (Jaffrey et al., 2001; Hess et al., 2005). S-nitrosylation of cysteine (Cys) is readily reversible with high spatial and temporal specificity. The NADH-dependent oxidoreductase, S-nitrosoglutathione reductase, specifically catalyzes the denitrosylation of S-nitrosoglutathione, by which protein S-nitrosylation is regulated in the cellular equilibrium between S-nitrosylated proteins and S-nitrosoglutathione. Thioredoxin also mediates direct denitrosylation of multiple S-nitrosylated proteins. Thus, the temporal and spatial regulation of S-nitrosylation and denitrosylation confers specificity to NO-based cellular signaling (Benhar et al., 2009).

**Figure 2:**
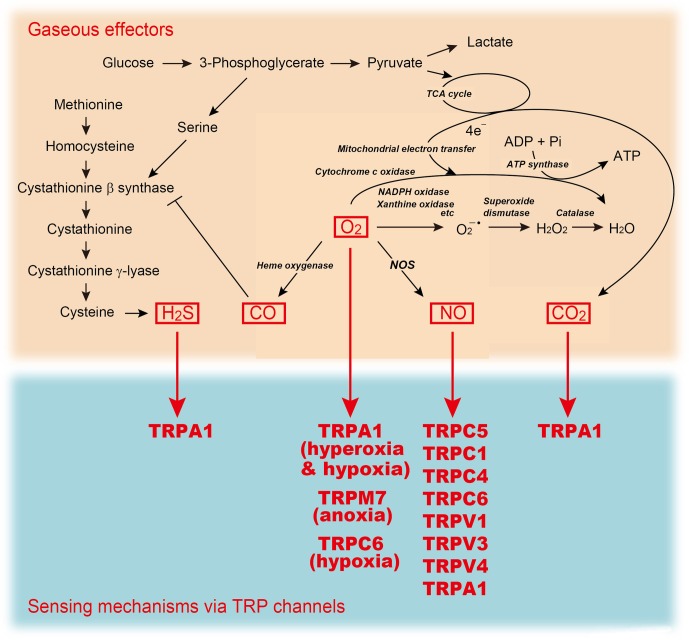
**A sensing mechanism for gaseous molecules linked to metabolic pathways.** TRP channels mediate sensing mechanism for H_2_S, O_2_ (anoxia, hypoxia, or hyperoxia), NO, and CO_2_.

Ca^2+^ and NO signals are precisely coordinated with each other and converge at two main points (Milbourne and Bygrave, 1995). Firstly, in order to be activated, constitutive NO synthase (NOS) must bind calmodulin (CaM), an event controlled by the level of [Ca^2+^]_i_ (Nathan, 1992). An increase in [Ca^2+^]_i_ has been shown to activate constitutive NOS, especially in endothelial and neuronal cells, and this results in NO production (Moncada et al., 1997). However, it is still unclear whether activation of endothelial NOS (eNOS) requires specific modes of upstream Ca^2+^-mobilization, or RACC subtypes formed by particular TRPCs (Hutcheson and Griffith, 1997; Lantoine et al., 1998; Lin et al., 2000; Koyama et al., 2002; Yao and Garland, 2005). In contrast, it is well characterized that Ca^2+^ influx through *N*-methyl-D-aspartate (NMDA) receptors activates neuronal NOS (nNOS) (Dawson et al., 1991) to elicit S-nitrosylation of many synaptic proteins (Jaffrey et al., 2001). The second point of convergence is that NO can directly affect intracellular Ca^2+^ levels by acting on cell surface receptors to promote Ca^2+^ mobilization (Milbourne and Bygrave, 1995). This feedback regulation of Ca^2+^ signaling by NO remains controversial in non-excitable cells (Khan and Hare, 2003), where NO has been reported to regulate Ca^2+^ mobilization pathways (including RACCs) both positively (Volk et al., 1997; Chen et al., 2000; Li et al., 2003) and negatively (Kwan et al., 2000; Dedkova and Blatter, 2002). In contrast, the regulation of Ca^2+^ signaling by NO is better defined in neurons, where NO nitrosylates NR1 and NR2 subunits of NMDA receptors at specific Cys/thiol groups and decreases Ca^2+^ entry *via* this ionotropic receptor (Choi et al., 2000; Jaffrey et al., 2001; Lipton et al., 2002). This negative feedback mechanism is important in precluding over-activation of NMDA receptors. However, NMDA receptors have a very restricted tissue distribution and specific physiological functions, whereas NO signaling *via* protein S-nitrosylation and Ca^2+^ signaling are much more generalized and have a wide array of functions. Therefore, to understand better the molecular mechanisms that link these two key signals, S-nitrosylation targets must be distinguished from the more ubiquitous Ca^2+^-mobilizing ion channels. As described below, this ultimately provides insights into the activation gating that underlies sensor function of TRP channels. In this review, we focus on how the gaseous signaling is sensed and integrated by TRP channels (Figure 2).

## Correlation between RACCs and NO signals in native tissue preparations

Tight correlation between native RACCs and NO signaling has been presumed in many types of cells (Xu et al., 1994; Volk et al., 1997; Ma et al., 1999; Chen et al., 2000; van Rossum et al., 2000; Thyagarajan et al., 2001; Li et al., 2003). In pancreatic acini isolated from rat, agonist-mediated Ca^2+^ release from internal stores activates a cellular pool of NOS to generate cGMP, which then modulates Ca^2+^ entry through the plasma membrane (Xu et al., 1994). This mechanism might be responsible for the capacitative nature of Ca^2+^ entry. Interestingly, different concentrations of an NO donor sodium nitroprusside (SNP) revealed that cGMP has a dual effect on Ca^2+^ entry. Increasing cGMP levels by up to 10-fold above that in control cells treated with *N*-nitro-L-arginine, a specific inhibitor of NOS, is associated with activation of Ca^2+^ entry. Further increases in cGMP to levels up to 80-fold above control inhibit Ca^2+^ entry in a concentration-dependent manner. The biphasic effect of cGMP provides the cells with a negative feedback mechanism and inhibits Ca^2+^ entry during periods of high [Ca^2+^]_i_, allowing oscillatory behavior in Ca^2+^ entry. In vascular endothelial cells isolated from bovine aorta, exogenous NO gas potentiates Ca^2+^ influx, which markedly increases the sustained phase of [Ca^2+^]_i_ elevation and possibly NO production (Chen et al., 2000). In DDT1MF-2 smooth muscle cell line derived from hamster vas deferens and DC-3F Chinese hamster lung fibroblast cell line, Ca^2+^ entry activated by the lipophilic NO donor, GEA3162 [5-amino-3-(3,4-dichlorophenyl)1,2,3,4-oxatriazolium] (25 μM), or the alkylator, N-ethylmaleimide (10 μM), is strongly activated by transient external Ca^2+^ removal that depletes internal Ca^2+^ stores, closely resembling activation of SOC activity in the same cells (Ma et al., 1999). In cortical astrocytes isolated from mice, physiological concentrations of a natural neuromessenger, ATP (10 μM), induces Ca^2+^-dependent NO production. By promoting Ca^2+^ influx, NO may facilitate the refilling of internal stores that have become partially depleted as a result of Ca^2+^ release during neurotransmitter-induced Ca^2+^ signaling (Li et al., 2003).

Suppressive effects of NO on Ca^2+^ entry have also been demonstrated (Kwan et al., 2000; Dedkova and Blatter, 2002). In vascular endothelial cells, the membrane permeant cGMP analogue 8-Br-cGMP (>300 μM) attenuates SOC activity *via* a protein kinase G (PKG)-dependent mechanism. These results suggest a role of cGMP and PKG in the regulation of Ca^2+^ entry in vascular endothelial cells (Kwan et al., 2000; Dedkova and Blatter, 2002). Contradictory effects of NO on Ca^2+^ influx may depend on the difference of the type of NO donors used for experiments and their concentrations. More importantly, the contradictory effects are at least in part attributable to diversity in the NO susceptibility of Ca^2+^ entry channels and signaling molecules that regulate Ca^2+^ influx. Thus, different ensembles of NO-regulated mechanisms may lead to a wide variety of net Ca^2+^ entry responses in the presence of NO.

## Regulation of TRP channels by NO *Via* Cys S-nitrosylation

**Figure 3:**
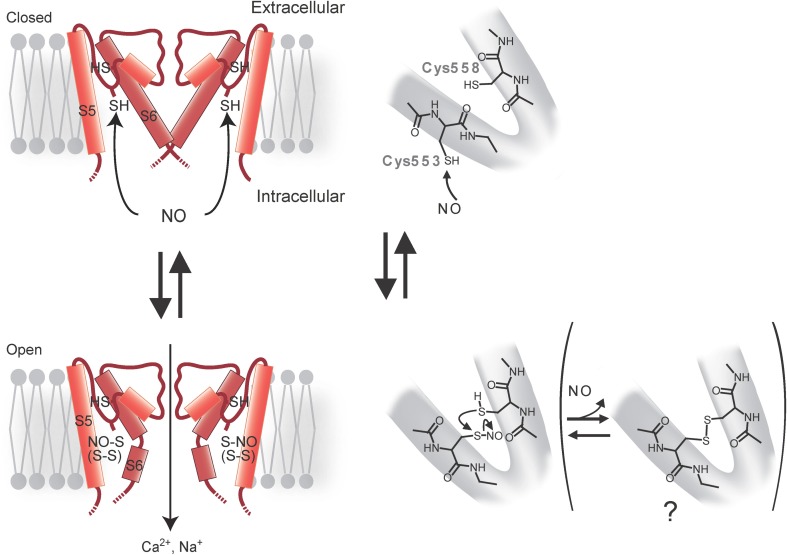
**Model for activation of TRPC5 channel by NO.** Cys553 is nitrosylated by NO, which triggers TRPC5 channel opening. The free sulfhydryl group of Cys558 nucleophilically attacks nitrosylated Cys553 to form a disulfide bond that stabilizes the open state.

Some TRP channels are potently regulated by Cys modifications, including Cys S-nitrosylation by NO in heterologous systems and bovine aortic endothelial cells (Yoshida et al., 2006). By performing labeling and functional assays with Cys mutants, it has been shown that Cys553 and nearby Cys558 on the N-terminal side of the putative pore-forming region between the fifth and sixth transmembrane domains S5 and S6 are essential for mouse TRPC5 activation in response to an NO donor, S-nitroso-N-acetyl-DL-penicillamine (SNAP) (300 μM) (Figure 3). In *Drosophila melanogaster* Shaker voltage-gated K^+^ channels, the activation gate formed by S6 residues near the intracellular entrance of the pore cavity has been identified (del Camino and Yellen, 2001). Considering the longer S5–S6 linkers of TRPC5, the TRPC5 S5–S6 linker with modified Cys553 and Cys558 may be invaginated toward the cytoplasm to reach the S6 activation gate (Figure 3). The corresponding Cys sites of TRPC1, TRPC4, TRPV1, TRPV3, and TRPV4 are potential targets of nitrosylation leading to channel activation in heterologous expression systems. Although the differences in maximal [Ca^2+^]_i_ responses [Δ ratio (340/380)] to SNAP, in control cells and cells heterologously expressing either TRPC1 or TRPC4β (a splice isoform of TRPC4) are not statistically significant, a larger fraction (7–9%) of the TRP-expressing cells shows a Δ ratio (340/380) >0.5 when compared to control cells (2–5%). Cells heterologously co-expressing TRPC4β and TRPC5 give responses to SNAP comparable to those in cells heterologously expressing TRPC5 alone, whereas cells heterologously co-expressing TRPC1 and TRPC5 give slightly suppressed yet robust responses. Co-immunoprecipitation of TRPC5 with TRPC1 or TRPC4β suggests that heteromultimeric TRPC5/TRPC1 and TRPC5/TRPC4β channels also have NO sensitivity. The thermosensor TRP channels TRPV1, TRPV3, and TRPV4 also show SNAP (300 μM)-induced activation, as predicted from conserved Cys residues in the corresponding regions of these homologs. Indeed, substitutions of two conserved Cys residues in TRPV1 lead to significantly suppressed responses to SNAP and to Cys S-nitrosylation. Notably, the sensitivity of TRPV1 to H^+^ and heat is enhanced by SNAP but abolished by the mutations, despite normal surface expression and intact control H^+^ and heat responses. Thus, channel activation regulated by nitrosylation is conserved among a number of TRP channels belonging to different subfamilies.

SNAP-activated TRPC5 channels are not entirely inactivated by ascorbate, which reduces S-nitrosothiols to thiols but not disulfides. However, dithiothreitol (DTT), which reduces both S-nitrosothiols and disulfides to thiols, fully suppressed SNAP-activated TRPC5 channel activity, suggesting that both nitrosylation and disulfide bond formation are involved in SNAP-induced TRPC5 activation. In an S-nitrosylation assay (Jaffrey et al., 2001), S-nitrosylation is abolished by mutation of Cys553 in TRPC5 but not by mutation of Cys558. As proposed for the acid–base catalysis of hemoglobin nitrosylation in proteins with high NO sensitivity, basic and acidic amino acids surrounding S-nitrosylated Cys may enhance the nucleophilicity of the sulfhydryl (SH) group, and therefore the S-nitrosylation of this group of proteins (Hess et al., 2005). Charged residues flanking Cys553 and Cys558 may confer modification susceptibility to NO in TRPC5. It is possible that the TRPC5 channel is opened *via* S-nitrosylation of Cys553 and a subsequent nucleophilic attack of nitrosylated Cys553 by the free SH group of Cys558 to form a disulfide bond that stabilizes the open state (Figure 3). The NO sensitivity of TRPC5 channels has been disputed by several groups (Xu et al., 2008; Wong et al., 2010). NO sensitivity of TRPC5 may depend on culturing conditions, the way drugs are administered, cell density during measurements, levels of antioxidants or other experimental conditions, molecular and cellular states that may affect the modification state of TRPC5 proteins.

**Figure 4:**
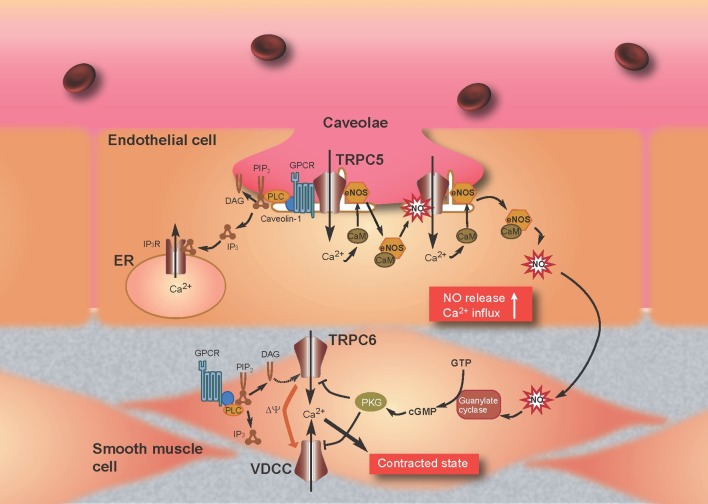
**Model for TRPC5-mediated feedback of Ca^2+^ and NO signaling in endothelial cells and attenuation of Ca^2+^ entry through TRPC6 by NO in smooth muscle cells.** Stimulation of G protein-coupled receptors (GPCRs) (such as the ATP-activated P2Y receptor) induces Ca^2+^ influx and activation of eNOS as a consequence of binding of Ca^2+^-CaM and release of eNOS from caveolin-1. TRPC5 undergoes eNOS-dependent S-nitrosylation after GPCR stimulation, resulting in amplified Ca^2+^ entry and secondary activation of eNOS to amplify production of NO. NO diffuses out of endothelial cells into adjacent smooth muscle cells and stimulates the guanylate cyclase, which leads to the activation of PKG in smooth muscle cells. In the most prevailing hypothesis, the magnitude of continuous Ca^2+^ influx through VDCC, which critically determines the contractile status of vascular smooth muscle cells, decreases and increases by membrane hyperpolarization and depolarization, respectively. TRPC6 likely functions as a depolarization (Δψ)-inducing channel or a direct Ca^2+^-entry pathway, activated in response to receptor stimulation. The NO/cGMP/PKG pathway suppresses TRPC6 and VDCC activity to induce relaxation of smooth muscle.

Previous reports have provided important information with respect to the formation in endothelial cells of a TRPC5 “channelsome,” a molecular assembly centered upon a channel. As mentioned above, TRPC1 forms heterotetrameric channels with TRPC5 (Strübing et al., 2001) and a protein complex with caveolin-1 in caveolae/lipid raft domains (Lockwich et al., 2000; Bergdahl et al., 2003), which regulate the plasma membrane trafficking of TRPC1 (Brazer et al., 2003). It is therefore possible that TRPC5 forms indirect protein complexes with caveolin-1 *via* TRPC1. Indeed, we have found an interaction of TRPC5 with caveolin-1 and eNOS in co-immunoprecipitation experiments as well as by colocalization of TRPC5 with caveolin-1 in heterologous systems and bovine aortic endothelial cells (Mori et al., unpublished data). Among numerous caveolin-associated proteins linked to signaling cascades (Couet et al., 1997; García-Cardeña et al., 1997; Sato et al., 2004; Quest et al., 2008), three isoforms of NOS, including eNOS, have been identified (Kone et al., 2003). The inhibitory association of caveolin is disrupted by the binding of Ca^2+^-CaM to eNOS, leading to eNOS activation (Ju et al., 1997; Michel et al., 1997a,b; Rizzo et al., 1998; Bernatchez et al., 2005). In addition, eNOS is activated by different kinases, including Akt, protein kinase A (PKA), and protein kinase C (PKC) (García-Cardeña et al., 1996; Fulton et al., 1999; Michell et al., 2001; Boo and Jo, 2003; Heijnen et al., 2004). Based on data from our and other laboratories, we can propose a plausible model to describe the role of the TRPC5 channelsome in regulating receptor-activated NO production in vascular endothelial cells (Figure 4). TRPC5 proteins form complexes with vasodilator receptors, G-proteins, PLCβ s and eNOS, and are anchored in caveolae by the scaffolding protein, caveolin-1. Upon vasodilator receptor stimulation, TRPC5 is activated by the PLCβ cascade to induce Ca^2+^ influx, which elevates the [Ca^2+^]_i_ to form Ca^2+^-CaM. This then releases eNOS from the inhibitory control of caveolin-1 and leads to an initial NO production that activates TRPC5 channels. Ca^2+^ influx *via* NO-activated TRPC5 channels then induces secondary activation of eNOS to amplify the production of NO, resulting in a positive feedback cycle of receptor-activated Ca^2+^ and NO signaling. This model has been neatly summarized in a short review by Stamler and colleagues (Foster et al., 2006), based largely on our own data (Yoshida et al., 2006).

Recent reports have shown that SNAP (30 μM) and another NO donor (6)-(E)-Ethyl-2-[(E)-hydroxyimino]-5-nitro-3-hexeneamide (NOR3) (300 μM) activate human TRPA1 in heterologous systems and mouse TRPA1 in dissociated sensory neurons (Sawada et al., 2008; Takahashi et al., 2008a; Miyamoto et al., 2009). Functional characterization of site-directed Cys mutants of TRPA1 has demonstrated that Cys421, Cys641, and Cys665 are responsible for human TRPA1 activation by NO (Takahashi et al., 2008a). Cys421, Cys641, and Cys665 are located within the eleventh AnkR domain, seventeenth AnkR domain, and the N-terminal cytoplasmic region between seventeenth AnkR domain and S1, respectively (Gaudet, 2008).

Injection of NOR3 (1.5 mM) in the hind paw after PLC and PKA pathway activation (which sensitizes nociceptors, including TRPV1 and TRPA1), causes nociceptive behavior (Miyamoto et al., 2009). Interestingly, a decrease in nociception is observed in mice lacking both TRPV1 and TRPA1, but not in individual knockout animals (Miyamoto et al., 2009) despite *in vitro* results showing that the NO donor can activate both ion channels. Note that due to solubility issues of NO donors, limitations exist in using NO donors at relatively high concentrations for behavioral experiments (<10-fold the *in vitro* EC_50_). For capsaicin, injections of capsaicin at >1000-times the *in vitro* EC_50_ are typically used to observe acute pain behavior. It is therefore not surprising that significant acute nocifensive behavior was not observed in terms of NO donors. Another potential explanation for the lack of a phenotype in individual knockout mice is due to functional compensation by each other. Indeed, TRPV1 and TRPA1 have overlapping expression in a subset of DRG neurons (Story et al., 2003) and thereby could potentially interact *via* intracellular signaling *in vivo*. Recently, icilin, an agonist of TRPA1 and TRPM8, has been implicated as a trigger for shaking and hyperthermia which require NO production and NMDA receptor activation (Ding et al., 2008; Werkheiser et al., 2009). In this context, it would be interesting to explore the mechanism of the concerted regulation of Ca^2+^ and NO signaling by the TRP channels, NMDA receptors and nNOS in neurons.

## Regulation of TRP channels *Via* the NO/cGMP/PKG pathway

One of the principal consequences of activation of the NO/cGMP/PKG cascade in blood vessels is vasorelaxation, which is mediated by phosphorylation of proteins that regulate intracellular Ca^2+^ levels and the sensitivity of the contractile machinery. Several distinct mechanisms have been proposed for the reduction in [Ca^2+^]_i_ caused by PKG (Lincoln et al., 2001; Feil et al., 2003). Increased activity of the BK_Ca_ channel following PKG activation has been reported to induce membrane hyperpolarization, thereby decreasing the rate of Ca^2+^ entry into vascular smooth muscle cells (VSMCs) through voltage-dependent Ca^2+^ channels (VDCCs). Phosphorylation of the inositol 1,4,5-trisphosphate receptor (IP_3_R)-associated protein, IRAG (IP_3_R-associated PKG substrate), is thought to inhibit agonist-induced Ca^2+^ release from internal stores. The possibility has also been suggested that phosphorylation of phospholamban by PKG increases the activity of sarcoplasmic Ca^2+^-ATPase, facilitating the active transport of Ca^2+^ into internal stores and thereby decreasing [Ca^2+^]_i_. In many different types of blood vessel, activation of PKG has been found to inhibit vasoconstrictor-induced Ca^2+^ influx through plasmalemmal pathways other than those employing VDCCs (Karaki et al., 1997). However, little information exists regarding the molecular components of such Ca^2+^ influx pathways and, accordingly, it remains unclear how phosphorylation *via* PKG activation reduces the rate of Ca^2+^ influx.

TRPC6 is a Ca^2+^-permeable channel regulated negatively by PKG-mediated phosphorylation in the NO/cGMP signaling pathway in heterologous systems and the rat vascular myocytes A7r5 (Takahashi et al., 2008b). Macroscopic and single-channel current recordings using patch clamp techniques have demonstrated that SNAP (100 μM)-induced inhibition of receptor-activated TRPC6 currents is abolished by pharmacological blockade of cGMP/PKG signaling with 1H-[1,2,4]oxadiazolo [4,3-a]quinoxalin-1-one (ODQ), 2,3,9,10,11,12-hexahydro-10R-methoxy-2,9-dimethyl-1-oxo-9S,12R-epoxy-1H-diindolo[1, 2,3-fg:3′,2′,1′-kl]pyrrolo[3,4-i][1,6]benzodiazocine-10-carboxylic acid methyl ester (KT5823) or membrane permeable PKG inhibitory peptide (DT3). It is also ablated by site-directed alanine mutation of a PKG phosphorylation site [threonine (Thr) 69] within the N-terminal cytoplasmic region of TRPC6. The critical involvement of Thr69 in PKG phosphorylation is confirmed by ^32^P-incorporation assays of wild-type and alanine-substitution mutant TRPC6 proteins. Similar NO/cGMP/PKG pathway-mediated negative regulation is also observed for TRPC6-like currents recorded in A7r5 VSMCs. Indeed, vasopressin-evoked membrane depolarization of these cells, which is expected secondarily to activate VDCCs, is significantly slowed and attenuated after application of SNAP (100 μM). The TRPC6 protein is abundantly expressed in various types of VSMCs and has been shown to be a constituent of vasoconstrictor-activated cation channels, which increase Ca^2+^ entry into VSMCs *via* direct Ca^2+^ permeation or secondary activation of a VDCC and/or Na^+^-Ca^2+^ exchanger (Inoue et al., 2006; Dietrich et al., 2007; Poburko et al., 2007). Thus, it is highly possible that, in a direct or indirect manner (i.e., *via* changes in membrane potential or an increase in intracellular Na^+^ concentration), PKG-mediated mechanisms may work as a universal negative feedback to regulate neurohormonal Ca^2+^ mobilization across the VSMC membrane (Figure 4). This mechanism may be physiologically important in vascular tissues where NO is constantly released from vascular endothelial cells or nitrergic nerves.

## O_2_ sensing mechanisms

Of the gaseous molecules, O_2_ is the most well-known to physicians, scientists and laymen alike as an essential physical requirement. O_2_ functions primarily as a terminal acceptor of electrons on mitochondrial electron transport. Most of the O_2_ consumed in this process is reduced to generate water through the actions of cytochrome oxidase. The remainder is used to generate compounds that exert potent biological actions, including prostaglandins, reactive oxygen species (ROS) and gaseous molecules such as NO and CO (Suematsu et al., 2003). In the last decade, O_2_ itself has been increasingly recognized as an important signal molecule that mediates many physiological and pathophysiological processes including proliferation of stem cells, ischemia injury and tumor progression (Csete, 2006; Swartz et al., 2007). Thus, O_2_ is not only required for cellular respiration, but also serves as a signaling molecule, and as the essential substrate for the formation of other signaling molecules (Figure 2). However, O_2_ also exerts toxicity causing aging, respiratory disorders and eventually death in a high O_2_ (hyperoxic) environment. Because of the ambivalent physiological nature of O_2_, aerobic life forms must adapt to hyperoxia and low O_2_ environment (hypoxia) by sensing O_2_ availability and transmitting this information to effector systems.

Cellular responses to changes in O_2_ availability can be acute or chronic (López-Barneo et al., 2001). Acute responses rely mainly on O_2_-regulated ion channels, which mediate adaptive changes in cell excitability, contractility, and secretory activity (Gonzalez et al., 1994; Neubauer and Sunderram, 2004; Weir et al., 2005). Chronic responses depend on the modulation of transcription factors such as hypoxia-inducible factor (HIF), which determines the expression of numerous genes encoding growth factors, enzymes, and transporters (Semenza and Wang, 1992; Schofield and Ratcliffe, 2004; Webb et al., 2009). O_2_-regulated ion channels and transcription factors are part of a widely operating signaling system that helps to provide an appropriate amount of O_2_ to the tissues and to protect the cells against toxicity damage due to excess or deficient O_2_.

In mammals, the carotid bodies, located near the carotid artery bifurcations, and brainstem catecholaminergic neurons rapidly detect changes in partial O_2_ pressure (*P*O_2_) in arterial blood (Gonzalez et al., 1994; Neubauer and Sunderram, 2004). It is known that BK_Ca_, TASK, and K_V_ K^+^ channels are involved in the mechanism of arterial O_2_ sensing in the carotid bodies (Gonzalez et al., 1994; Weir et al., 2005). Hypoxia inhibits K^+^ channels through several mechanisms, such as CO production by hemeoxygenase and intracellular ATP depletion that depolarizes glomus cells. This inhibition leads to activation of VDCC, exocytosis, and the excitation of carotid sinus nerves. In contrast, hyperoxia attenuates depolarization and inhibits exocytosis (Gonzalez et al., 1994; Weir et al., 2005). The chemosensory inputs of the carotid sinus nerve are carried toward the medullary centers that regulate the ventilatory pattern. The local O_2_ tension is also rapidly detected by other tissues including vagal and sensory neurons in the airway and lungs, chromaffin cells of the fetal adrenal medulla, smooth muscle cells of the pulmonary resistance arteries, cerebral neurons, fetoplacental arteries, systemic arteries, and the doctus arteriosus. However, detection of hypoxia by these tissues remains to be fully characterized. Recently, a class of TRP channels has been demonstrated to act as cell sensors for changes in O_2_ availability (Aarts et al., 2003; Weissmann et al., 2006; Takahashi et al., 2011).

### Anoxia-sensing mediated by TRPM7 channels in the brain

Excitotoxicity in brain ischemia triggers neuronal death and neurological disability, and yet these are not prevented by antiexcitotoxic therapy (AET) in humans. The failure of AET in the face of a clear role for excitotoxicity in acute neurological disorders is paradoxical (Birmingham, 2002; Ikonomidou and Turski, 2002). In addressing this problem, Aarts et al. have revealed that the TRPM7 channel, termed chanzyme because it possess a channel and α-kinase domain (Runnels et al., 2001; Nadler et al., 2001), is activated by oxygen-glucose deprivation (OGD) through the production of ROS and RNS, permitting Ca^2+^ uptake that further stimulates ROS and TRPM7 activation in heterologous systems and cortical neurons isolated form rats (Aarts et al., 2003). Suppressing TRPM7 expression in rat cortical neurons prevents anoxic neuronal death even in the absence of AET, indicating that TRPM7 is an essential mediator of anoxic death. It is possible that patients enrolled in failed trials studying the use of AET for stroke or traumatic brain injury are selected to have severe injuries (Morris et al., 1999), or that these disorders in humans, by their nature, induce severe ischemia. Therefore, future treatment of such disorders may also need to inhibit TRPM7. Indeed, it has been shown that suppression of hippocampal TRPM7 by intrahippocampal injections of viral vectors bearing shRNA specific for TRPM7 makes neurons resistant to ischemic death after brain ischemia and preserves neuronal morphology and function in rats (Sun et al., 2009). TRPM7 suppression also prevents ischemia-induced deficits in long-term potentiation and preserves performance in fear-associated and spatial-navigational memory tasks. Thus, regional suppression of TRPM7 is feasible, well tolerated and inhibits delayed neuronal death *in vivo* in an animal model.

### Hypoxia-sensing by TRPC6 channels in pulmonary smooth muscle cells

Regional alveolar hypoxia causes local vasoconstriction in the lungs, shifting blood flow from hypoxic to normoxic areas, thereby maintaining gas exchange. This mechanism is known as hypoxic pulmonary vasoconstriction (HPV) (Jeffery and Wanstall, 2001; Weissmann et al., 2001; Schermuly et al., 2005). Disturbances in HPV can cause life-threatening hypoxemia, whereas chronic hypoxia triggers vascular remodeling in the lungs and pulmonary hypertension (Sartori et al., 1999). In studying signaling cascades of this vitally important mechanism, Weissmann et al. have shown that severe hypoxia (1% O_2_)-induced cation influx and currents in smooth muscle cells are largely absent in precapillary pulmonary arteries of *Trpc6* knockout mice, although recombinant TRPC6 expressed heterologously cannot be activated by hypoxia (Weissmann et al., 2006). Hypoxia-induced TRPC6 activation in smooth muscle cells is mediated by DAG accumulation probably by activated phospholipases. TRPC6 appears to be a key regulator of acute HPV, because this regulatory mechanism is absent in *Trpc6* knockout mice, whereas the pulmonary vasoconstrictor response to the thromboxane mimetic, U46619, is unchanged. Accordingly, induction of regional hypoventilation results in severe arterial hypoxemia in *Trpc6* knockout mice, but not in wild type mice. Notably, chronic hypoxia-induced pulmonary hypertension is independent of TRPC6 activity. Thus, TRPC6 plays a unique and indispensable role in acute HPV. Manipulation of TRPC6 function may thus offer a therapeutic strategy for the control of pulmonary hemodynamics and gas exchange.

### O_2_-sensing by TRPA1 channel in vagal and sensory neurons

Sensory and vagal afferent neurons, which project nerve endings throughout the body, are thought to detect hypoxia in organs such as the airway, lungs and heart after ischemia, and other conditions of low O_2_ supply (Howe et al., 1981; De Sanctis et al., 1991; Longhurst et al., 2001; Gruss et al., 2006). However, the characteristics and mechanisms of hypoxia detection by sensory and vagal neurons have yet to be fully defined (Longhurst et al., 2001). In terms of hyperoxia, *Caenorhabditis elegans* has been reported to be adept at avoiding hyperoxia due to detection mechanisms in sensory neurons (Gray et al., 2004). Furthermore, insects breathe discontinuously to avoid O_2_ toxicity during hyperoxia (Hetz and Bradley, 2005). However, the physiological relevance of hyperoxia detection through sensory systems is less clear in vertebrates, and the delineation of these neuronal hyperoxia-sensing molecular processes in vertebrates remains an exciting area of research.

**Figure 5:**
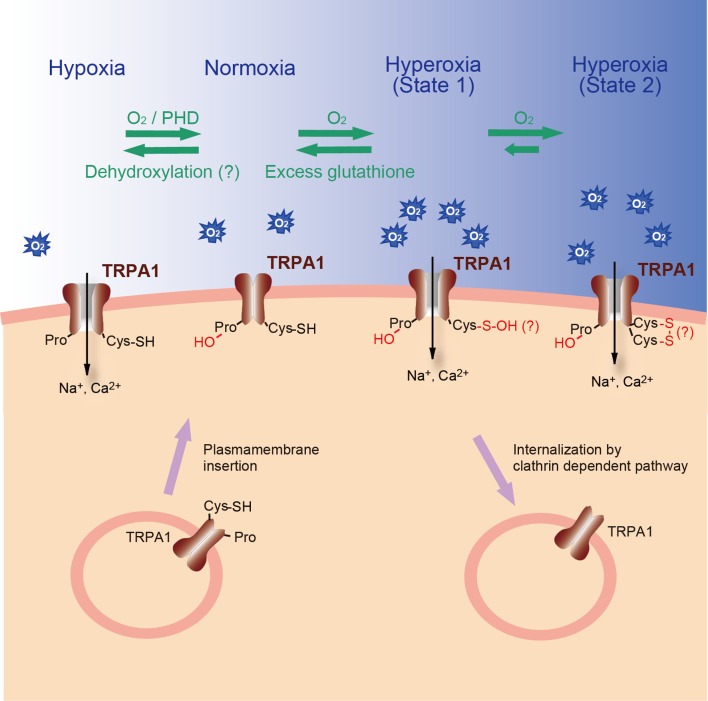
**Model for O_2_-sensing of TRPA1 channel.** PHDs hydroxylate specific Pro residue on the N-terminal AnkR domain of TRPA1 protein in normoxia, whereas a decrease in O_2_ concentrations diminishes PHD activity and relieves TRPA1 from the hydroxylation, leading to its activation in hypoxia. The relief can be achieved by insertion of unmodified TRPA1 proteins into the plasma membrane or by dehydroxylation through an unidentified molecular mechanism. In hyperoxia, O_2_ oxidizes specific Cys residues, thereby activating TRPA1. TRPA1 may at least take two oxidized state upon hyperoxia: a relatively unstable oxidized state (state 1) readily reversed by glutathione and a relatively stable oxidized state (state 2). Sulfhydryl group(s) of the key Cys residues may be modified to sulfenic acid in the former state of TRPA1, whereas that in the latter state of TRPA1 may form a disulfide bond(s). These oxidation mechanisms over-ride the inhibition by Pro hydroxylation to activate TRPA1.

Systematic evaluation of TRP channels using reactive disulfides with different redox potentials has revealed that TRPA1 can sense O_2_ (Takahashi et al., 2011). O_2_ sensing by TRPA1 is based upon disparate processes: proline (Pro) hydroxylation by Pro hydroxylases (PHDs), and direct oxidation of Cys residues. In normoxia, PHDs hydroxylate conserved Pro394 within the tenth AnkR domain of TRPA1, inhibiting its activity. In hypoxia, the decrease in O_2_ concentration diminishes PHD activity, relieving TRPA1 from the inhibitory action of Pro hydroxylation and leading to its activation. This recovery of TRPA1 activity can be achieved by insertion of fresh, unmodified TRPA1 proteins into the plasma membrane or by dehydroxylation of modified proteins through an unidentified molecular mechanism. In hyperoxia, O_2_ activates TPRA1 by oxidizing Cys633, Cys856 or both. Cys633 and Cys856 are located within the seventeenth AnkR domain and the intracellular linker region between S4 and S5, respectively. TRPA1 can assume at least two oxidized forms during hyperoxia: a relatively unstable oxidized state (state 1) readily reversed by glutathione, and a relatively stable oxidized state (state 2). SH groups on the key Cys residues (Cys633 and Cys856) may be modified to sulfenic acid (S-OH) in state 1, and form disulfide bonds (S-S) in state 2. This oxidation mechanism over-rides the inhibition by Pro hydroxylation to activate TRPA1. In mice, disruption of the *Trpa1* gene abolishes hyperoxia- and mild hypoxia (15% O_2_)-induced cationic currents in vagal and sensory neurons and thereby impedes enhancement of *in vivo* vagal discharges induced by hyperoxia and hypoxia. The results suggest a new O_2_-sensing mechanism mediated by TRPA1 (Figure 5).

The vagal nerve conveys sensory information about the state of the organs to the central nervous system, in addition to providing output to the various organs in the body. Enhanced discharges in vagal afferents induce respiratory, cardiac, and vascular responses (Meller and Gebhart, 1992; Longhurst et al., 2001; Kubin et al., 2006). Chemicals encountered in the airway are detected by airway vagal C fibers (Kubin et al., 2006). Recently, TRPA1 has been shown to sense environmental irritants, thus initiating defensive reflexes such as coughing and respiratory depression in the C fibers (Bessac and Jordt, 2008; Bessac et al., 2008; Nassenstein et al., 2008). Notably, TRPA1 activation shows an inverted bell-shaped O_2_-dependency curve with a minimum at the *P*O_2_ of 137 mmHg, which is slightly below the atmospheric *P*O_2_ (159 mmHg) (Takahashi et al., 2011). Considering that tracheal *P*O_2_ (149 mmHg) is comparable to atmospheric *P*O_2_ (Cottrell, 1988), it is highly possible that TRPA1 expressed in the trachea is slightly but significantly activated to act as a frontline defense against mild hyperoxia in the atmosphere.

Weather records (McElroy, 2002) suggest that atmospheric *P*O_2_ at sea level ranges between approximately 137 and 170 mmHg. Because our O_2_ dependence data reveal that minimal TRPA1 activity at a *P*O_2_ of 137 mmHg is approximately 30% of the maximum activity at a *P*O_2_ of 170 mmHg, so-called normoxia can be hyperoxic in the context of mammalian TRPA1 channels. This is reminiscent of O_2_ avoidance in *Caenorhabditis elegans* (Gray et al., 2004) and insects (Hetz and Bradley, 2005).

## Sensing of other gaseous molecules by TRP channels

H_2_S has emerged as a new gaseous modulator of various biological functions including nociception (Szabó, 2007; Gadalla and Snyder, 2010). In rodents, topical application of an H_2_S donor, NaHS (1 nmol) produces pain responses through potentiation of T-type Ca^2+^ channels probably in the primary afferents (Kawabata et al., 2007; Matsunami et al., 2009). Endogenous H_2_S is also reported to contribute to pain transmission in rat models of formalin-induced inflammation (Lee et al., 2008) and irritable bowel syndrome (Xu et al., 2009).

NaHS (1 mM) activates capsaicin-sensitive sensory neurons in isolated rat urinary bladder (Patacchini et al., 2005). Although the mechanisms underlying the action of H_2_S are yet to be clarified, the pharmacological profile of H_2_S hints at the involvement of TRPA1. Indeed, Streng et al. have shown that NaHS (1 mM) causes activation of human and mouse TRPA1 in heterologous expression systems, suggesting that TRPA1 is indeed a molecular target for H_2_S in the bladder (Streng et al., 2008). In this context, it is interesting to note that bladder inflammation can be triggered by TRPA1 activation (Cox, 1979; McMahon and Abel, 1987) and that potential pathogens, such as *Escherichia coli*, can produce H_2_S (Berglin and Carlsson, 1985). Recently, Miyamoto et al. have shown that NaHS (1 μM)-evoked increases in [Ca^2+^]_i_ are inhibited by removal of external Ca^2+^ and by a TRPA1-specific inhibitor, HC-030031, suggesting that H_2_S stimulates sensory neurons *via* activation of TRPA1 (Miyamoto et al., 2011). Furthermore, the hyperalgesia/allodynia induced in mice by intraplantar administration of NaHS (100 pmol) is significantly suppressed by pre-administration of a TRPA1-specific blocker AP18 and by silencing TRPA1 channels in sensory neurons (Okubo et al., 2012). Thus, H_2_S-induced mechanical hyperalgesia and allodynia require activation of TRPA1 channels.

In humans, gaseous CO_2_ produces a pungent sensation, as noted by the Scottish philosopher Alexander Bain more than 100 years ago (Cain and Murphy, 1980). A variety of sensory structures and receptors mediate the responses to CO_2_ in different organisms (Luo et al., 2009). For example, in flies, gaseous CO_2_ is detected by gustatory receptors on the antenna, whereas dissolved CO_2_ is detected on the proboscis: CO_2_ is either aversive or attractive depending on the sensory structure activated (Suh et al., 2004; Fischler et al., 2007; Jones et al., 2007; Kwon et al., 2007). In mice, ingested CO_2_ is sensed by taste receptors in the mouth (Chandrashekar et al., 2009), and blood CO_2_ is detected in the brainstem by K^+^ channels (Trapp et al., 2008) and in the amygdala by acid-sensing ion channel (ASIC) 1a (Ziemann et al., 2009). Atmospheric levels of CO_2_ are detected by guanylyl cyclase D, which is expressed in a subset of olfactory sensory neurons in mice (Luo et al., 2009). However, this latter system is missing in humans and most other primates (Young et al., 2007). It is widely believed that the noxious sensation of CO_2_ is due to activation of the trigeminal nerve fibers that innervate the nasal and oral cavities (Silver and Moulton, 1982; Steen et al., 1992). Recently, Wang et al. have shown that CO_2_ specifically activates a subpopulation of trigeminal neurons expressing a functional *Trpa1* gene (Wang et al., 2010). CO_2_-induced activation of TRPA1 is downstream of intracellular acidification, consistent with our observation that TRPA1 is activated by H^+^ (Takahashi et al., 2008a). Thus, TRPA1 makes an important contribution to nociceptive responses to CO_2_.

## Conclusions

TRP channels respond to multiple activation triggers and therefore serve as polymodal signal detectors. An important aspect of this multimodal activation of TRP channels is their role in signal integration and amplification. When a TRP channel is activated by downstream or upstream constituents (molecules/proteins/enzymes in a specific signaling cascade), in addition to the primary activation trigger immediately upstream, the TRP channel plays an important role in forming positive feedback or feed-forward loops in the cascade. For example, in vascular endothelial cells, Ca^2+^ influx *via* NO-activated TRPC5 channels can amplify production of NO by eNOS, resulting in the enhancement of NO production in nearby endothelial cells and NO-dependent relaxation of smooth muscle cells. In smooth muscle cells, NO exerts inhibitory effects on TRPC6 channel activity through PKG-mediated phosphorylation. Together, these mechanisms are capable of ensuring the fidelity of cellular responses and minimizing variation in their magnitude, synchronizing the responses of neighboring cells that comprise functional domains within vascular tissues to reduce blood pressure and maintain local blood flow.

The accumulating evidence summarized above strongly suggests that TRPV1 and TRPA1 are sensors that transduce gaseous signals into electrical signals in sensory and vagal neurons. The chemosensory inputs to these neurons are propagated toward the central nervous system to induce pain sensation or to change ventilatory patterns. However, the roles of Ca^2+^ influx *via* TRPV1 and TRPA1 in controlling Ca^2+^ signaling pathways in neurons remain elusive. Considering that the PHD-HIF pathway is central to chronic hypoxia responses that increase red blood cell mass and stimulate new blood vessel growth (Semenza and Wang, 1992; Webb et al., 2009), it would be interesting to examine the effect of Ca^2+^ influx through O_2_-sensitive TRPA1 channels on the functional regulation of PHD and HIF. Studies of TRP channels have been extended dramatically from the simple functional description of single molecules to the holistic analysis and integration of the molecular systems controlled by TRP channels.

### Conflict of interest statement

The authors declare that the research was conducted in the absence of any commercial or financial relationships that could be construed as a potential conflict of interest.
